# Evaluation of the expression stability of reference genes in *Apis mellifera* under pyrethroid treatment

**DOI:** 10.1038/s41598-020-73125-w

**Published:** 2020-09-30

**Authors:** Przemysław Wieczorek, Patryk Frąckowiak, Aleksandra Obrępalska-Stęplowska

**Affiliations:** Department of Molecular Biology and Biotechnology, Institute of Plant Protection-National Research Institute, Władysława Węgorka 20 St, 60-318 Poznań, Poland

**Keywords:** RNA metabolism, Transcription, Entomology

## Abstract

Honeybees (*Apis mellifera* L.), which unquestionably play an economically important role in pollination and agricultural production, are at risk of decline. To study changes in gene expression in insects upon exposure to pesticides or other external stimuli, appropriate reference genes are required for data normalization. Since there is no such gene that is absolutely invariable under all experimental conditions, the aim of this study was to identify the most stable targets suitable for subsequent normalization in quantitative experiments based on real-time polymerase chain reaction in honeybee research. Here, we evaluated the expression of fifteen candidate housekeeping genes from three breeding lines of honeybees treated with pyrethroids to identify the most stable genes. The tested insects were exposed to deltamethrin or lambda-cyhalothrin, and then, changes in the accumulation of selected transcripts were assessed, followed by statistical analyses. We concluded that *AmRPL32*, *AmACT* and *AmRPL13a* were the commonly recorded most stable genes in honeybees treated with the selected pyrethroids.

## Introduction

The importance of honeybees (*Apis mellifera* L.) as pollinators is unquestionable. However, since intensive crop production is currently strictly dependent on pesticide use, honeybees are exposed to agrochemicals during pollination^1^, and these chemicals seem to impact the insects.

Pyrethroids are an important group of insecticides used for insect pest management^2^. They are the most frequently used crop protection products for crops pollinated by honeybees, and their residues are most often found in these insects^3–5^. Pyrethroids target the nervous system of the treated individuals; their active compound binds to voltage-gated sodium channels (VGSCs) in neurons and alters the function of these channels by maintaining the channel opening on axonal membranes. As a result, the neuron membranes cannot repolarize, leaving the axonal membrane permanently depolarized, thereby paralysing the organism^6^. By performing gene expression analyses, it is possible to understand how honeybees react to pyrethroids at the molecular level. As a result, the data obtained are then relevant for sustainability programmes. For this purpose, several methods can be used, such as next-generation sequencing (RNAseq), microarrays, northern hybridization or quantitative reverse transcription polymerase chain reaction (RT-qPCR). Because of its versatility, low cost and high detection rate, RT-qPCR is now not only a very important tool for a majority of gene expression studies but also a standard for validating results derived from RNAseq^7^ or microarray analyses^8^ in which differentially expressed genes are studied. However, similar to other RNA-based quantitative techniques, RT-qPCR experiments need to be carefully designed and performed^9^. The experimental design is based on, among other factors, the selection of a good internal control, that is, a gene that exhibits stable expression under the experimental conditions being tested. Only by using this approach is it possible to accurately estimate the accumulation of target mRNA molecules. However, it should be noted that there is no universal gene that might be used for RT-qPCR normalization under every experimental condition. The expression of housekeeping genes (HKGs) can be influenced by many factors; therefore, validation of their stability should always be performed before quantitation of mRNA targets^10,11^. The need of careful selection of reference genes for gene expression studies in insects was widely reviewed previously^12^.

In this study, we analysed the stability of the expression of fifteen genes, used previously in selection of reference genes in insects^12^, involved in basic metabolism of the honeybees, namely, actin (*AmACT*), α-tubulin (*AmTUB*), glutathione-S-transferase (*AmGST1*), glyceraldehyde-3-phosphate dehydrogenase (*AmGAPDH*), porphobilinogen deaminase (*AmHMBS*), ribosomal protein L32 (*AmRPL32*), 60S ribosomal protein L13a (*AmRPL13a*), 40S ribosomal protein S18 (*AmRP18S*), succinate dehydrogenase (*AmSDHA*), TATA-box-binding protein (*AmTBP*), elongation factor 1-alpha (*AmEF1α*), arginine kinase (*AmARGK*), chitin synthase 6 (*AmCHS6*), dorsal (*AmDORS*), and 18S ribosomal RNA (*Am18S*), in *A. mellifera* L. exposed to two types of pyrethroids: deltamethrin and lambda-cyhalothrin. The aim of the study was to identify the HKGs stably expressed in honeybees. We performed experiments to determine the best reference genes (a) for all the experimental conditions tested (namely, for all the breeding lines under treatment with pyrethroids), (b) for a particular breeding line individually, and (c) by focusing on two different active pyrethroid compounds. By utilizing RT-qPCR and four statistical algorithms, we concluded that regardless of the conditions tested, the genes *AmRPL32*, *AmACT* or *AmRPL13a* were commonly found among the most stable genes in honeybees treated with the mentioned pyrethroids. Moreover, by performing pairwise variation analysis (V_*n/n*+*1*_), we determined that two of the identified reference genes would be sufficient for accurate normalization of RT-qPCR experimental results. Finally, we validated the results and used the selected reference genes to measure the expression of two cytochrome P450 monooxygenase (*AmCYP450*) genes described previously to be influenced in honeybees treated with insecticides^13,14^, and therefore the expression of *AmCYP6AQ1*^15^ and *AmCYP305a1*^13^ assayed in the research.

## Results

### Determination of the specificity of the designed primers

In this study, we evaluated fifteen candidate genes from honeybees to check their stability in insects exposed to pyrethroid treatment (Table 1). The main goal of the research was to identify the most stable genes that could be used as internal controls in experiments based on RT-qPCR to determine or verify differentially expressed genes in honeybees treated with pyrethroids.

**Table 1:** List of primers used in the study.

Target name	Sequence of used oligonucleotides 5′ → 3′				
	Forward primers	Reverse primers	Accession number	Estimated length in RT-PCR [bp]	Calculated Tm after RT-qPCR [°C]
Actin	AmAct_F TGCCAACACTGTCCTTTCTG	AmAct_R AGAATTGACCCACCAATCCA	AB023025.1	156	79.89
Tubulin alpha-1	AmTub_F AATCGGCAAAGAAATTGTCG	AmTub_R TACCACCACCGAATGAGTGA	XM_396338.6	107	78.82
Glutathione-S-transferase 1	AmGST_F ACGCTTACCGTTGCTGATTT	AmGST_R CCCGTTCATCAAATTGACCT	AY620822.2	174	83.43
Glyceraldehyde-3-phosphate dehydrogenase 2	AmGAPDH_F TGCTCAGGTTGTTGCCATTA	AmGAPDH_R CAGCTCCAGCTTTTGTCCAT	XM_393605.6	197	75.91
Porphobilinogen deaminase	AmHMBS_F AAAAGCGAGTTGGCTCTGAA	AmHMBS_R AAATCAACACGGCCACTTTC	XM_624258.5	197	75.46
Ribosomal protein L32	AmRPL32_F TGTGCTGAAATTGCTCATGG	AmRPL32_R CGTAACCTTGCACTGGCATA	NM_001011587.1	104	77.81
60S ribosomal protein L13a	AmRPL13a_F TGGCCATTTACTTGGTCGTT	AmRPL13a_R GAGCACGGAAATGAAATGG	XM_623810.5	191	77.51
40S ribosomal protein S18	AmRP18S_F GATTCCCGATTGGTTTTTGA	AmRP18S_R CCCAATAATGACGCAAACCT	XM_625101.5	149	76.79
Succinate dehydrogenase [ubiquinone] flavoprotein subunit	AmSDHA_F GGCAAAGCTGCAAAAATCTC	AmSDHA_R AAGCTGCACGTAATCCTGCT	XM_623062.5	109	79.15
TATA-box-binding protein	AmTBP_F TGATCGGAACACCACAAAAA	AmTBP_R AAGCCGGTGTCATAGGTGTC	XM_623085.5	189	78.67
Elongation factor 1-alpha F2	AmEF1a_F TGATGCTCCTGGACACAGAG	AmEF1a_R GAAATGCCTGCTTCGAACTC	XM_006569890.3	114	78.27
Arginine kinase	AmARGK_F GTGCACATCAAGCTGCCTAA	AmARGK_R GATTCCATCGTGCATCTCCT	NM_001011603.1	192	82.89
Chitin synthase 6	AmCHS6_F GGAGCACATGATTGGTTGTG	AmCHS6_R CGATCTTCCCCTTGATCGTA	XM_001123000.3	150	78.55
18S ribosomal RNA	Am18S_F CGCACGAGATTGAGCAATA	Am18S_R TCCTCGTTCATGGGGAATAA	AY703484.1	170	84.22
Dorsal (transcription factor)	AmDorsal_F TCGGATGGTGCTACGAGCGA	AmDorsal_R AGCATGCTTCTCAGCTTCTGCCT	NM_001011577	153	79.59
Cytochrome P450 (*CYP6AQ1*)	AmCYP450_R TGCATCGGTATGCGACTAGG	AmCYP450_R AAGAGTTTAACCAGCCGCGA	NM_001205062	192	78.72
Cytochrome P450 305a1 (*CYP305a1*)	AmCYP450_305a1_F TCGATCTTTTTCTCGCTGGT	AmCYP450_305a1_R TTGCTTTGTCCTCCATGTTG	XM_623618.6	156	77.24

**Figure 1:**
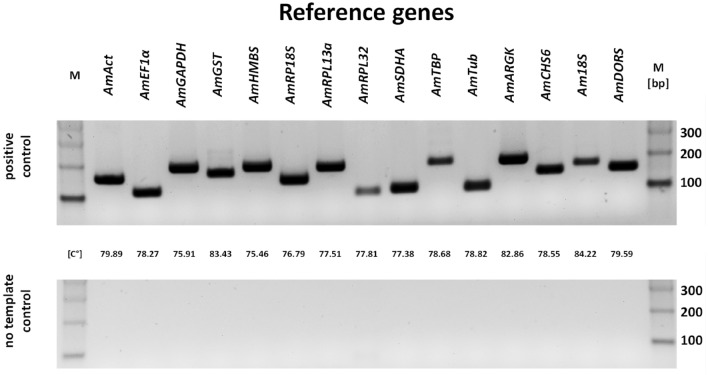
Amplification products of end-point RT-PCR performed with primers designed for RT-qPCR. *M* DNA molecular weight ladder, *bp* base pairs.

First, from the GenBank database, we retrieved cDNA sequences of *A. mellifera* L. encoding these genes and used the data as input in Primer3 for designing the best primer pairs. By doing so, fifteen primer pairs that matched the implemented parameters were chosen, with the resulting amplicon lengths between 100 and 250 bp and the annealing temperature of the primers set at approximately 60 °C. Then, by performing a pilot experiment (end-point RT-PCR), we tested the designed primers for their specificity. The products of the RT-PCR were resolved on an agarose gel, and after staining, a single DNA band was detected for each tested primer pair (Fig. 1). No amplification products were detected in the no-template control reactions. Moreover, the results from Sanger sequencing of the cloned amplicons verified the sequence specificity of the primers used.

**Figure 2:**
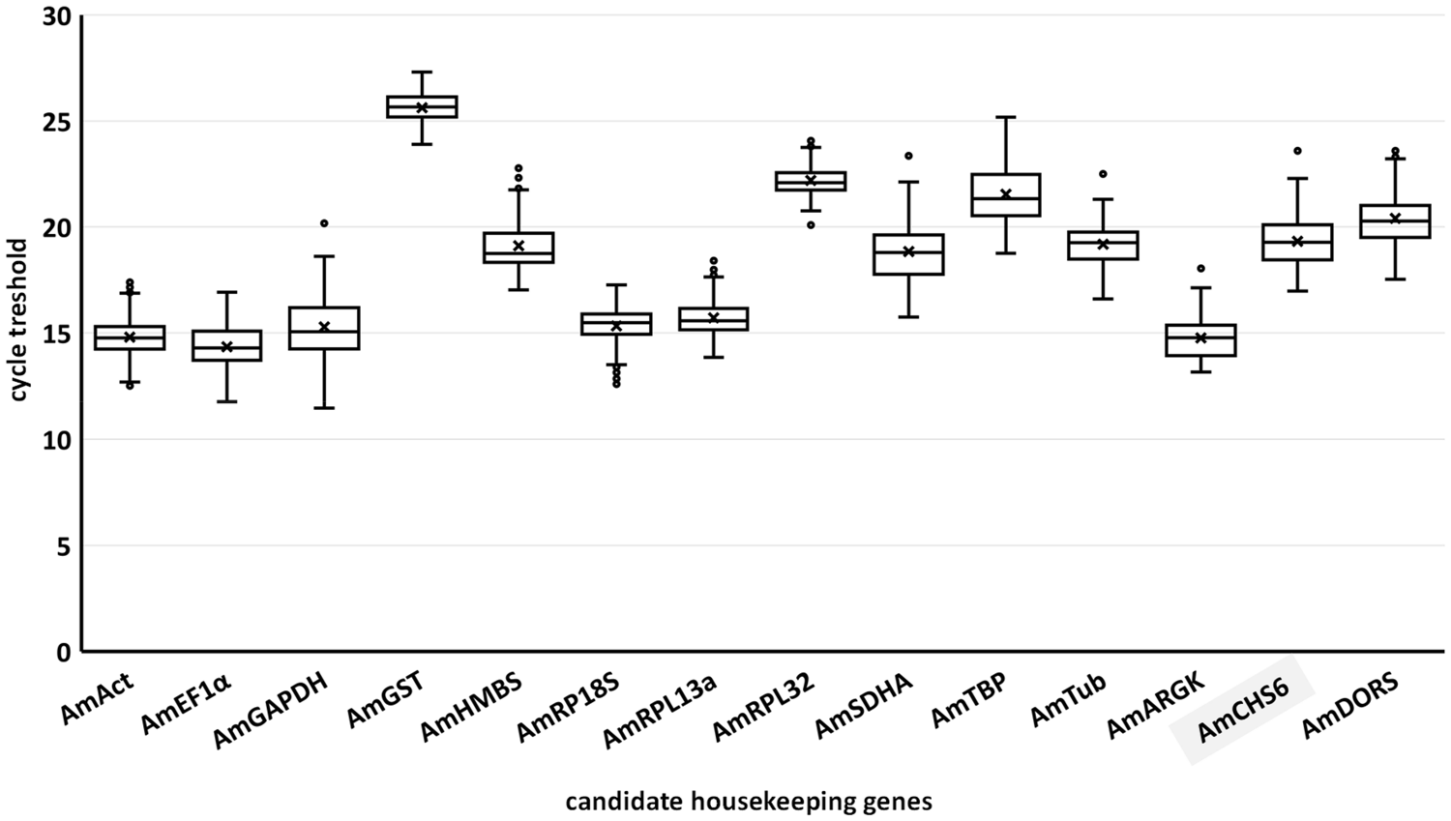
Box plot indicating the distribution of C_T_ values after RT-qPCR for each primer pair. The C_T_ values were considered across all tested samples (n = 108).

**Figure 3:**
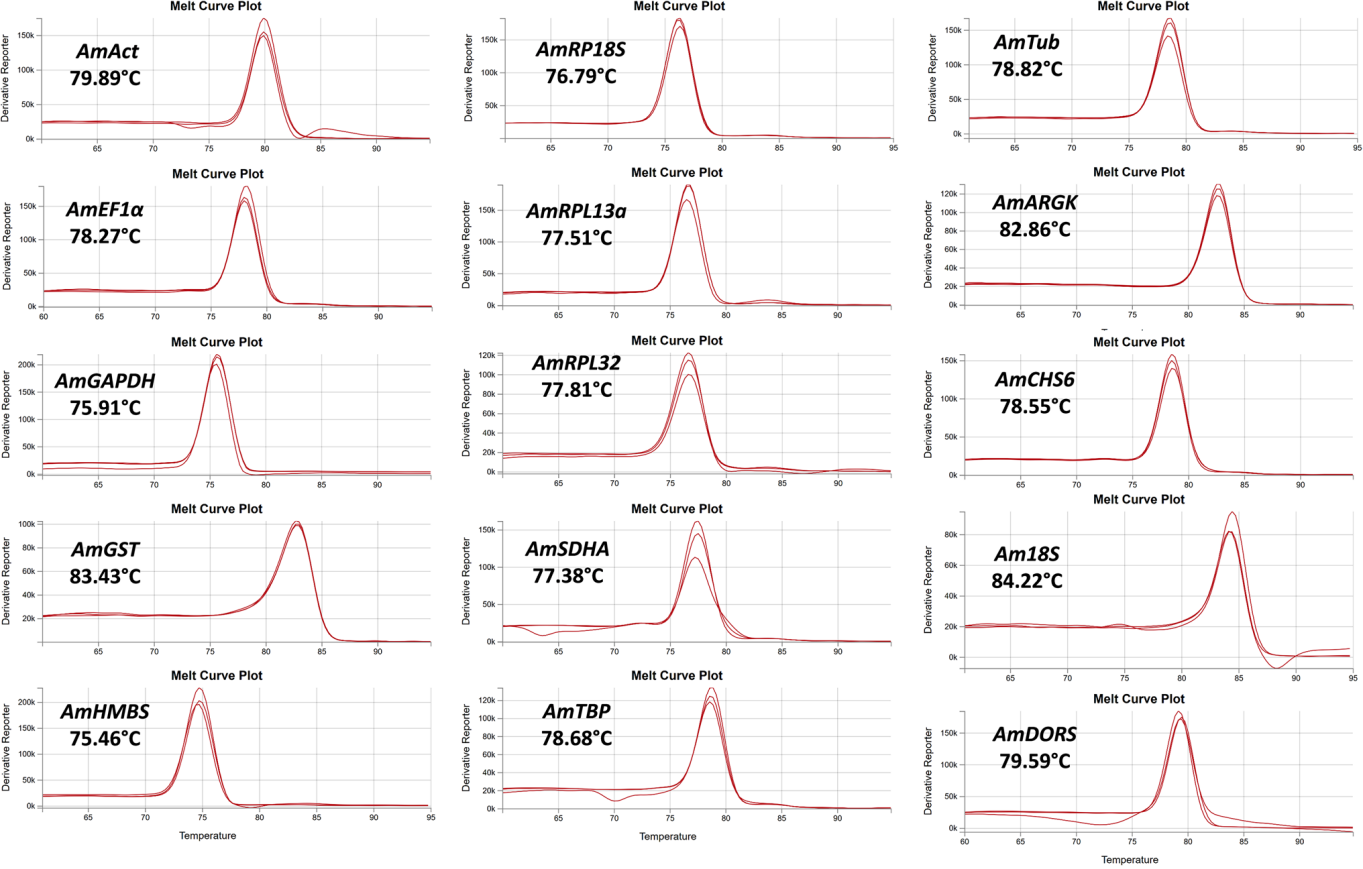
Melting plots generated after RT-qPCR. The fifteen primer pairs were evaluated by quantitative RT-PCR. The melting temperature is indicated on each plot.

Next, we examined the expression rates of the tested transcripts: after each RT-qPCR, all the C_T_ output data were grouped in a table, and a combined box plot was prepared. All of the fifteen tested genes were amplified by RT-qPCR. The highest expression rate was observed for *Am18S* rRNA, with a C_T_ value of approximately 5.61. However, we omitted *Am18S* rRNA in further analyses because of the extremely high accumulation of rRNA in the analysed insects. The C_T_ values of all other candidate genes ranged between 14.33 and 25.62, which were the values for *AmEF1α* and *AmGST*, respectively (Fig. 2). Importantly, the expression levels of the analysed genes were similar among the three tested breeding lines (see Supplementary Fig. S1). Next, analysis of melting curves generated during the melting stage of RT-qPCR verified the presence of a single amplification product in each reaction: the generated melting curves were sharp and symmetric (Fig. 3), indicating reaction specificity. The melting temperature of the amplicons ranged from 75.46 to 84.33 °C, which were the values for *AmHMBS* and *Am18S*, respectively.

### Stability analysis of candidate reference genes

In the present study, we focused on fourteen candidate HKGs of *A. mellifera* L. treated with two pyrethroids: deltamethrin and lambda-cyhalothrin. Our general goal was to identify the most stable reference genes in honeybees (a) regardless of breeding line and chemical compounds used for treatment and (b) individually for three breeding lines, and (c) for the two pyrethroids used for treating the insects. To achieve this goal, we used the following statistical tools: geNorm, BestKeeper, NormFinder and ΔCT. The comprehensive analysis was performed using RefFinder (https://www.heartcure.com.au/reffinder).

#### Search for the most stable reference genes for all breeding lines exposed to pyrethroids

geNorm software, installed as a Bioconductor “NormqPCR” package in R software^16^, was used as the first program to identify the stability of the predicted HKGs, and the resulting values were used to order the HKGs from the most stable (the lowest M value) to the least stable (the highest M value). The *AmHMBS* gene and the *AmSDHA* gene had the highest expression stability values (0.0597 and 0.0604 M, respectively), followed by *AmTub*, *AmTBP,* and *AmRPL32*. The five least stable genes were *AmRPL13a, AmGST, AmRPL18S, AmGAPDH* and *AmEF1α* (Table 2).

**Table 2:**
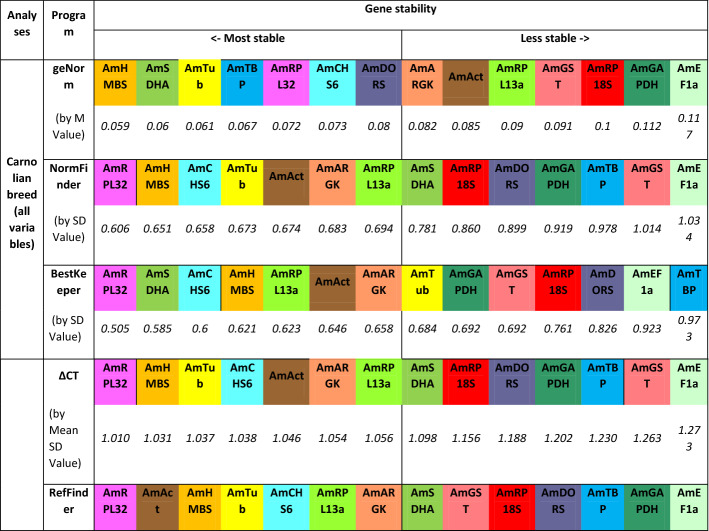
Stability ranking of fourteen candidate reference genes in *Apis mellifera* L. Carnolian honeybees under pyrethroid treatment.

The calculations were performed by geNorm, NormFinder, BestKeeper, ΔCT and RefFinder.

Next, three additional methods were used to calculate the most stable reference genes within the pool of all tested samples. NormFinder analysis classified the *AmRPL32, AmHMBS, AmCHS6, AmTub* and *AmAct* genes as the most stable genes, whereas the *AmEF1α, AmGST, AmTBP, AmGAPDH* and *AmDORS* genes were indicated as being the least stable. The ΔCT analysis showed that *AmRPL32, AmHMBS, AmTub, AmCHS6* and *AmAct* had the highest expression stability in comparison to *AmEF1α, AmGST, AmTBP, AmGAPDH* and *AmDORS*, which exhibited unstable expression. Then, based on the standard deviation (SD) of the C_T_ measurements, the stability values for the expression of fourteen candidate reference genes were calculated using the BestKeeper program, which showed slight differences compared to previous algorithms. The *AmRPL32, AmSDHA, AmCHS6, AmHMBS,* and *AmRPL13a* genes were identified as the most stable genes, and the *AmTBP, AmEF1α, AmDORS, AmRP18S* and *AmGST* genes were identified as the least stable genes.

Finally, to prepare a general ranking of most stable/unstable genes, a comprehensive analysis was performed with the support of RefFinder. According to the recommended comprehensive ranking, the *AmRPL32, AmAct, AmHMBS, AmTub,* and *AmCHS6* genes were identified as the five most stable genes, and the *AmRP18S, AmDORS, AmTBP, AmGAPDH,* and *AmEF1α* genes were identified as the five least stable genes.

#### Analysis of HKG stability among the three breeding lines

Next, the mentioned calculating methods were implemented to check the most stable HKGs in honeybees with regard to their breeding origin (breeding line) separately.

For the Kortówka line (Table 3), the five most stable genes were as follows: *AmGST, AmRPL32, AmTub, AmARGK* and *AmAct* (according to geNorm); *AmAct, AmARGK, AmRPL13a, AmCHS6* and *AmSDHA* (according to NormFinder); *AmGST, AmARGK, AmRPL32, AmTub* and *AmAct* (according to BestKeeper); *AmAct, AmARGK, AmRPL13a, AmCHS6* and *AmSDHA* (according to the ΔCT method).

**Table 3:**
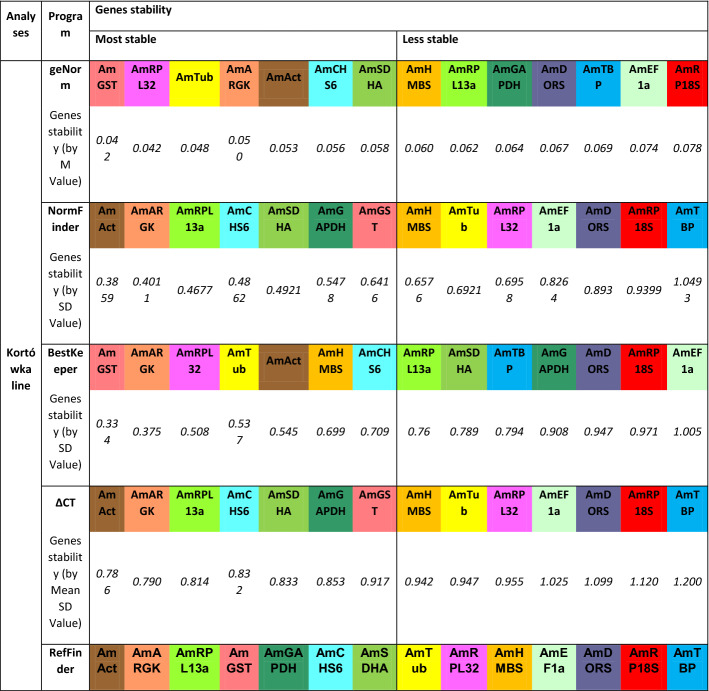
Stability ranking of fourteen candidate reference genes in the *Apis mellifera* L. Kortówka breeding line under pyrethroid treatment.

The calculations were performed by geNorm, NormFinder, BestKeeper, ΔCT and RefFinder.

For the Kortówka breeding line, the RefFinder method ordered the most stable genes as follows: *AmAct*, *AmARGK*, *AmRPL13a*, *AmGST* and *AmGAPDH*.

For the Alpejka line (Table 4), the most stable genes were Am*TBP, AmDORS, RPL32, AmHMBS* and *AmRP18S* (according to geNorm); *AmDORS, AmRP18S, AmEF1α, AMHMBS* and *AmRPL13a* (according to NormFinder); *AmDORS, AmRP18S, AmRPL32, AmGST* and *AmRPL13a* (according to BestKeeper); and *AmDORS, AmRP18S, AmEF1α, AmHMBS* and *AmRPL13a* (according to the ΔCT method).

**Table 4:**
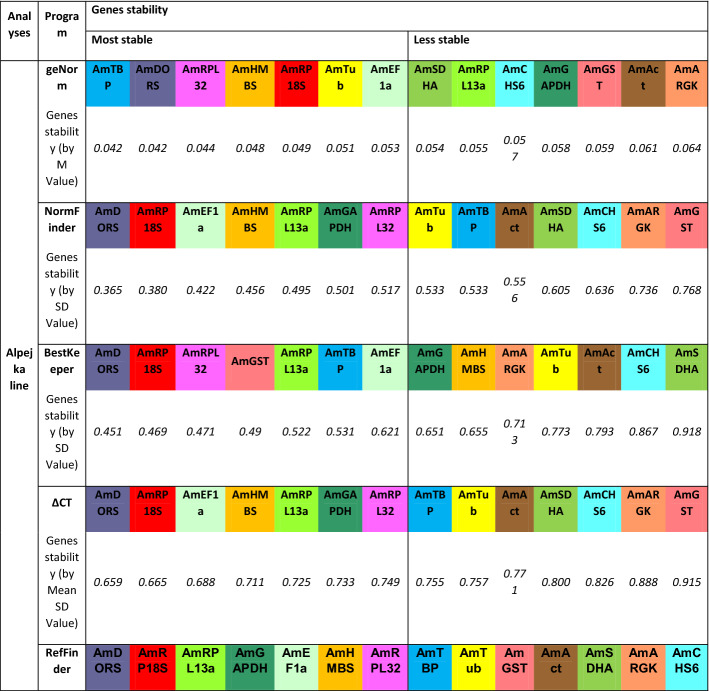
Stability ranking of fourteen candidate reference genes in the *Apis mellifera* L. Alpejka breeding line bred under pyrethroid treatment.

The calculations were performed by geNorm, NormFinder, BestKeeper, ΔCT and RefFinder.

On the basis of the abovementioned results, the RefFinder analysis identified *AmDORS*, *AmRP18S*, *AmRPL13a*, *AmGAPDH*, and *AmEF1α* as the most stable genes for the Alpejka breeding line.

For the Nieska line (Table 5), the most stable genes were *AmTBP, AmDORS, AmRPL32, AmHMBS, AmRP18S* (according to geNorm); *AmARGK, AmTub, RPL13a, AmCHS6* and *AmTBP* (according to NormFinder); *AmRPL32, AmRP18S, AmRPL13a, AmAct* and *AmGST* (according to BestKeeper); and *AmARGK, AmTub, AmRPL13a, AmRP18S* and *AmRPL32* (according to the ΔCT method).

**Table 5:**
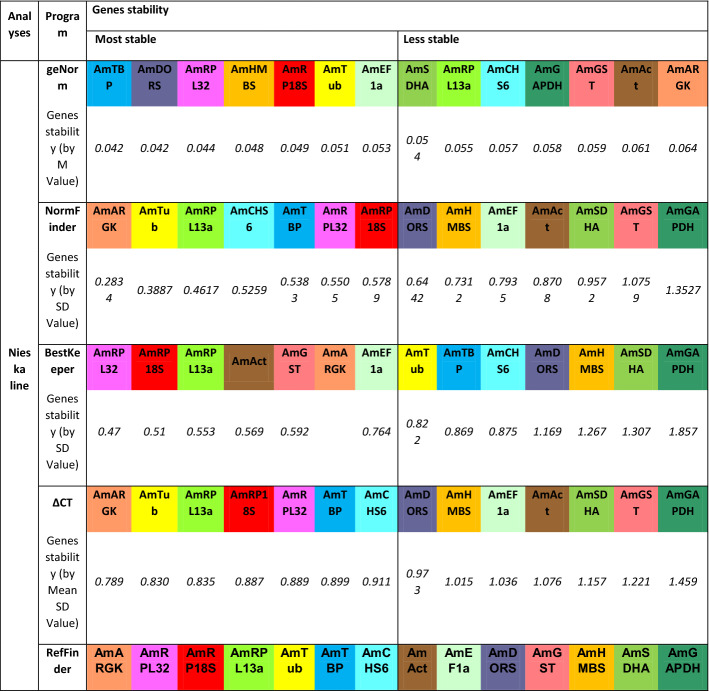
Stability ranking of fourteen candidate reference genes in the *Apis mellifera* L. Nieska breeding line under pyrethroid treatment.

The calculations were performed by geNorm, NormFinder, BestKeeper, ΔCT and RefFinder.

Thus, the following genes were selected as the most stable genes according RefFinder calculations for the Nieska line: *AmARGK*, *AmRPL32*, *AmRP18S*, *AmRPL13a* and *AmTub* (Table 5).

#### Analysis of the HKG stability with regard to the active substance of pyrethroid insecticide

Analysis of the influence of each insecticide used for insect treatment on HKG stability was also performed (Table 6). All calculations were performed as stated above using geNorm, NormFinder, BestKeeper, ΔCT and RefFinder. In insects treated with deltamethrin, the rank order of the most stable genes was as follows: *AmSDHA*, *AmTub*, *AmHMBS*, *AmTBP* and *AmRPL32* (according to the geNorm method); *AmCHS6*, *AmRPL32*, *AmARGK*, *AmHMBS* and *AmRPL13a* (according to NormFinder); *AmRPL32*, *AmRPL13a*, *AmGST*, *AmAct* and *AmRP18S* (according to the BestKeeper); *AmCHS6, AmRPL32*, *AmTub*, *AmHMBS* and *AmARGK* (according to the ΔCT method).

**Table 6:**
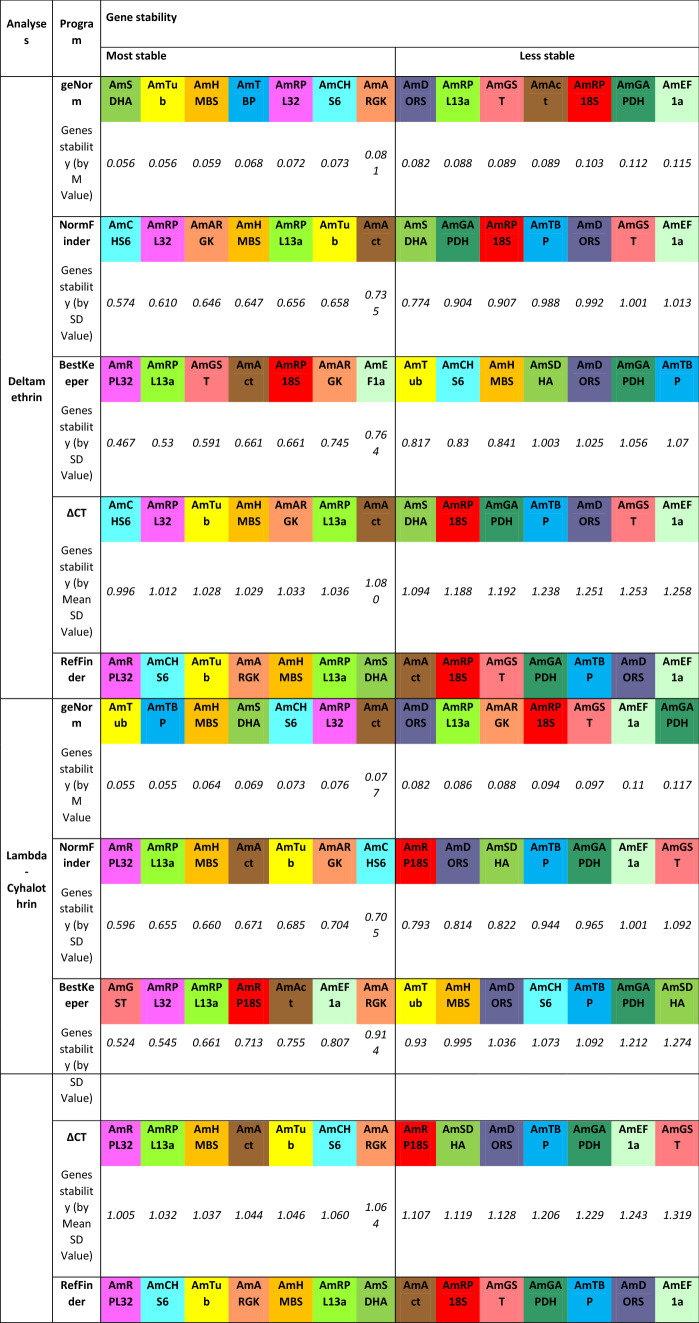
Stability ranking of fourteen candidate reference genes in *Apis mellifera* L. under pyrethroid treatment.

The calculations were performed by geNorm, NormFinder, BestKeeper, ΔCT and RefFinder.

The comprehensive analysis performed by RefFinder ranked the most stable genes in the following order for deltamethrin treatment: *AmRPL32*, *AmCHS6*, *AmTub*, *AmARGK* and *AmHMBS*.

The same calculations were performed to select HKGs stably expressed in *A. mellifera* L. exposed to lambda-cyhalothrin. The C_T_ data obtained after RT-qPCR were grouped in a table and subjected to subsequent calculations. The geNorm method indicated the following genes as being the most stable: *AmTub*, *AmTBP*, *AmHMBS*, *AmSDHA* and *AmCHS6*. Next, the list of the five most stable genes calculated by NormFinder included *AmRPL32*, *AmRPL13a*, *AmHMBS*, *AmAct* and *AmTub*. BestKeeper selected *AmGST*, *AmRPL32*, *AmRPL13a*, *AmRP18S* and *AmAct* as the most stable genes. *AmRPL32*, *AmRPL13a*, *AmHMBS*, *AmAct* and *AmTub* were identified as the most stable genes using the ΔCT method.

Finally, comprehensive analysis (by RefFinder) indicated *AmRPL32*, *AmCHS6*, *AmTub*, *AmARGK* and *AmHMBS* as the most stable genes under lambda-cyhalothrin exposure.

### Determination of the minimum number of reference genes necessary for normalization

**Figure 4:**
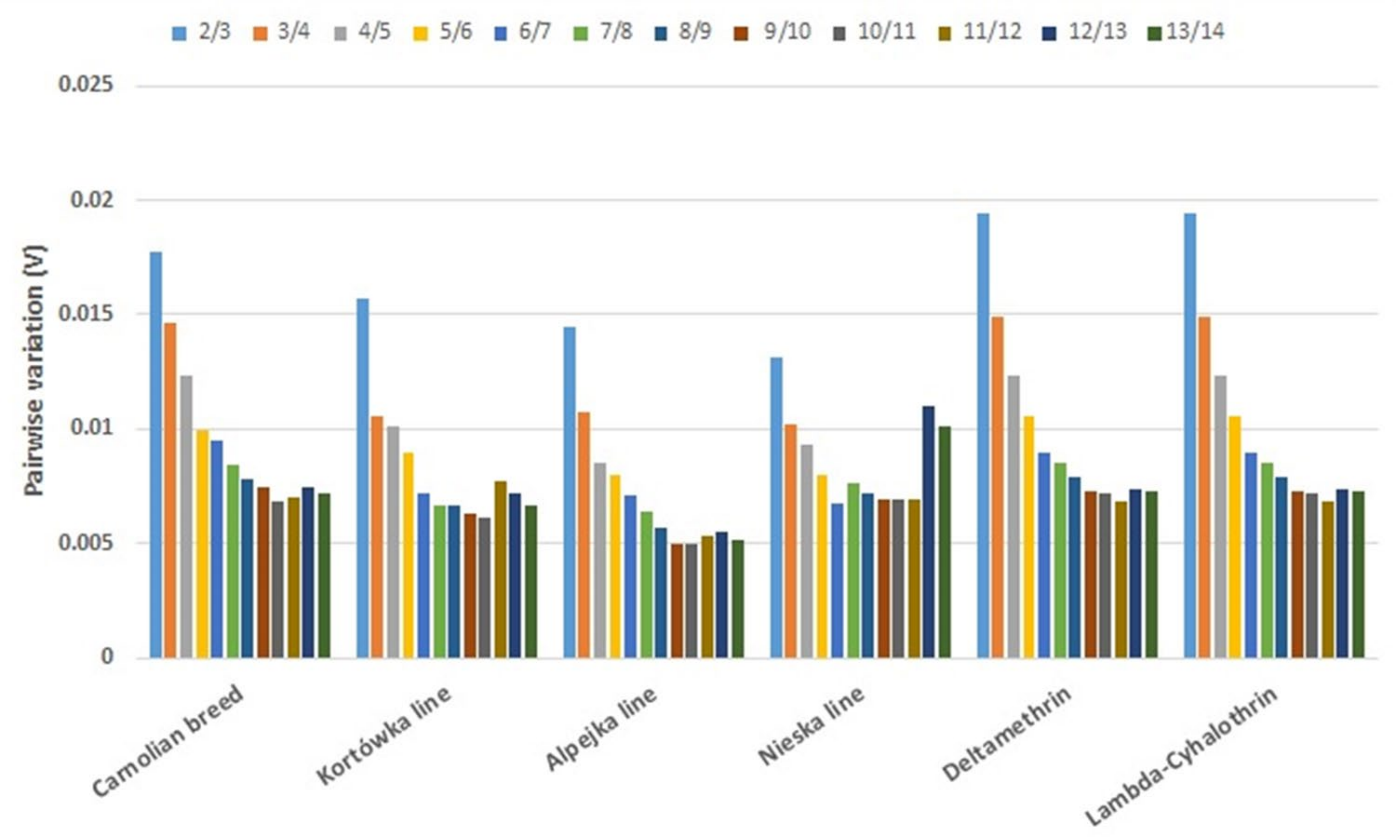
Optimal number of reference genes for various conditions. The geNorm algorithm was used to determine the pairwise variation (V) between the reference genes for treatments with pyrethroids together (Carnolian honeybees) or separately (deltamethrin or lambda-cyhalothrin). The effect of pyrethroid treatments on three breeding lines was also indicated (Kortówka, Alpejka and Nieska). The threshold for adequate normalization was V ≤ 0.15.

Pairwise variation analysis (V_*n/n*+*1*_) performed using the geNorm method^17^ indicated that the expression of the target gene in each considered experimental variant needs to be normalized using two selected reference genes. This was indicated by pairwise variation (V) with the threshold value set at 0.15^17^. In all tested experimental variants, the V_2/3_ value was lower than 0.15 (Fig. 4).

### Validation of reference genes

To validate the obtained results (the indicated stable HKGs for each experimental condition), we performed an analysis of the expression of two cytochrome P450 monooxygenase (*AmCYP450*) genes in honeybees exposed to pyrethroid treatment. CYP450s are known to be involved in xenobiotic detoxification in insects^14^. Importantly, it was described previously that the expression of *AmCYP6AQ1*^15^ and *AmCYP305a1*^13^ was influenced by insecticides. Validation experiments aimed at normalization of the expression of both *AmCYP450* genes were performed in the following contexts: first, for the entire set of tested Carnolian honeybees exposed to pyrethroid treatment; second, for testing the effect of pyrethroid treatment on the expression of *AmCYP450* in each breeding line separately; and finally, for analysing the expression of *AmCYP450* separately in deltamethrin- or lambda-cyhalothrin-treated insects.

As indicated earlier in the manuscript, using two reference genes is sufficient for accurate normalization of genes in pyrethroid-treated insects. For normalization of *AmCYP450* expression in Carnolian honeybees, the two following HKGs were used: *AmRPL32* and *AmHMBS*.

**Figure 5:**
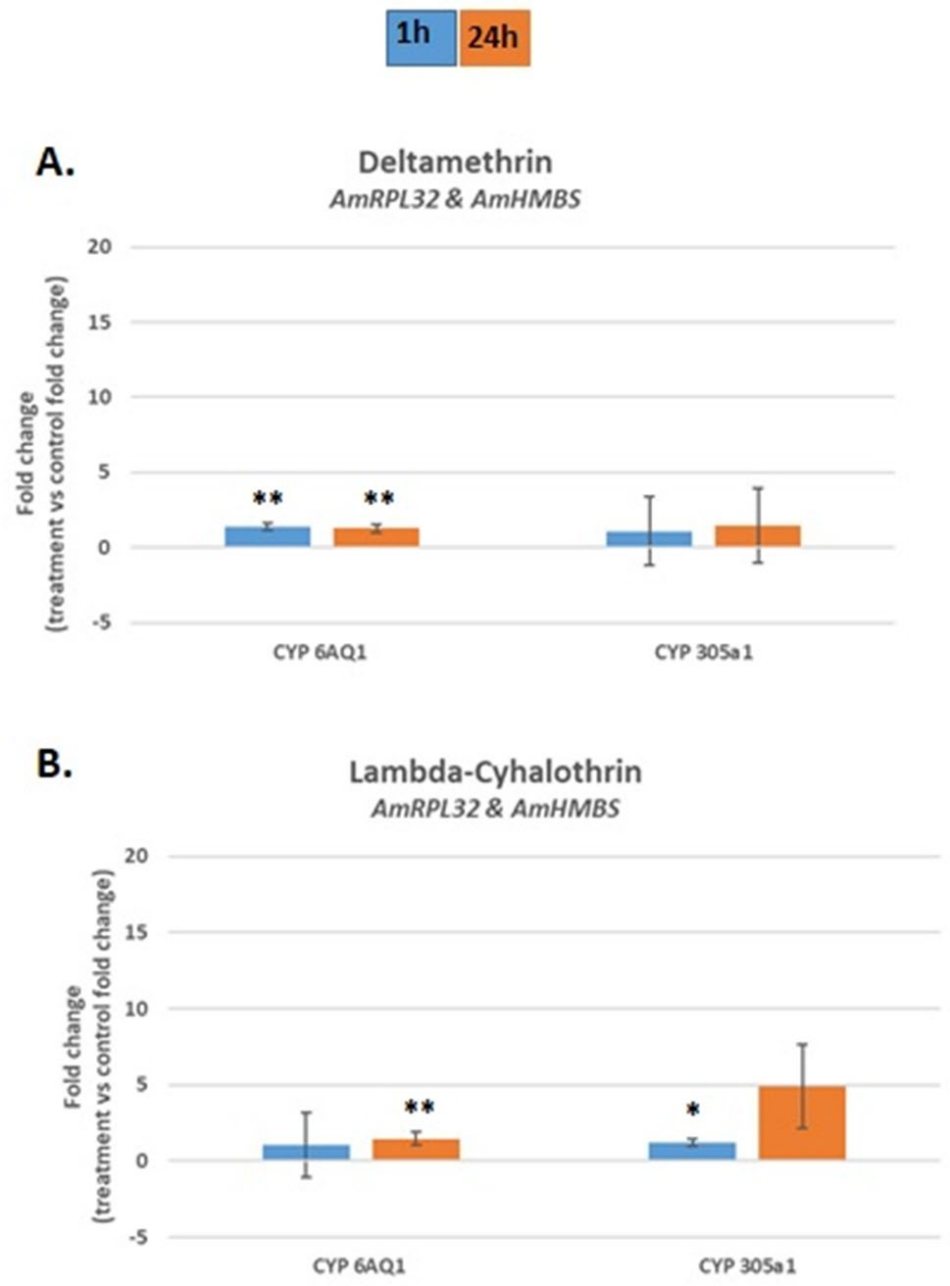
Expression of the two *AmCYP450* genes *Am*C*YP6AQ1* and *AmCYP305a1* in *Apis mellifera* L. treated with either deltamethrin (**A**) or lambda-cyhalothrin (**B**) normalized against the indicated reference genes (*AmRPL32* and *AmHMBS*). Blue bars: 1 h post treatment, orange bars: 24 h post treatment. Error bars represent the standard deviation. The Mann–Whitney U-test was used. **p < 0.01, *p < 0.05.

The results showed (Fig. 5) that expression of the *AmCYP6AQ1* gene increased slightly in honeybees treated with deltamethrin (1 h and 24 h after treatment) and lambda-cyhalothrin (24 h after treatment) with a 1.35-fold change, 1.28-fold change and 1.47-fold change (all with p < 0.01), respectively. On the other hand, the expression of *AmCYP305a1* in pyrethroid-treated honeybees increased over time, reaching a 4.91-fold change 24 h after treatment (p > 0.05) in the insects exposed to lambda-cyhalothrin (Fig. 5).

**Figure 6:**
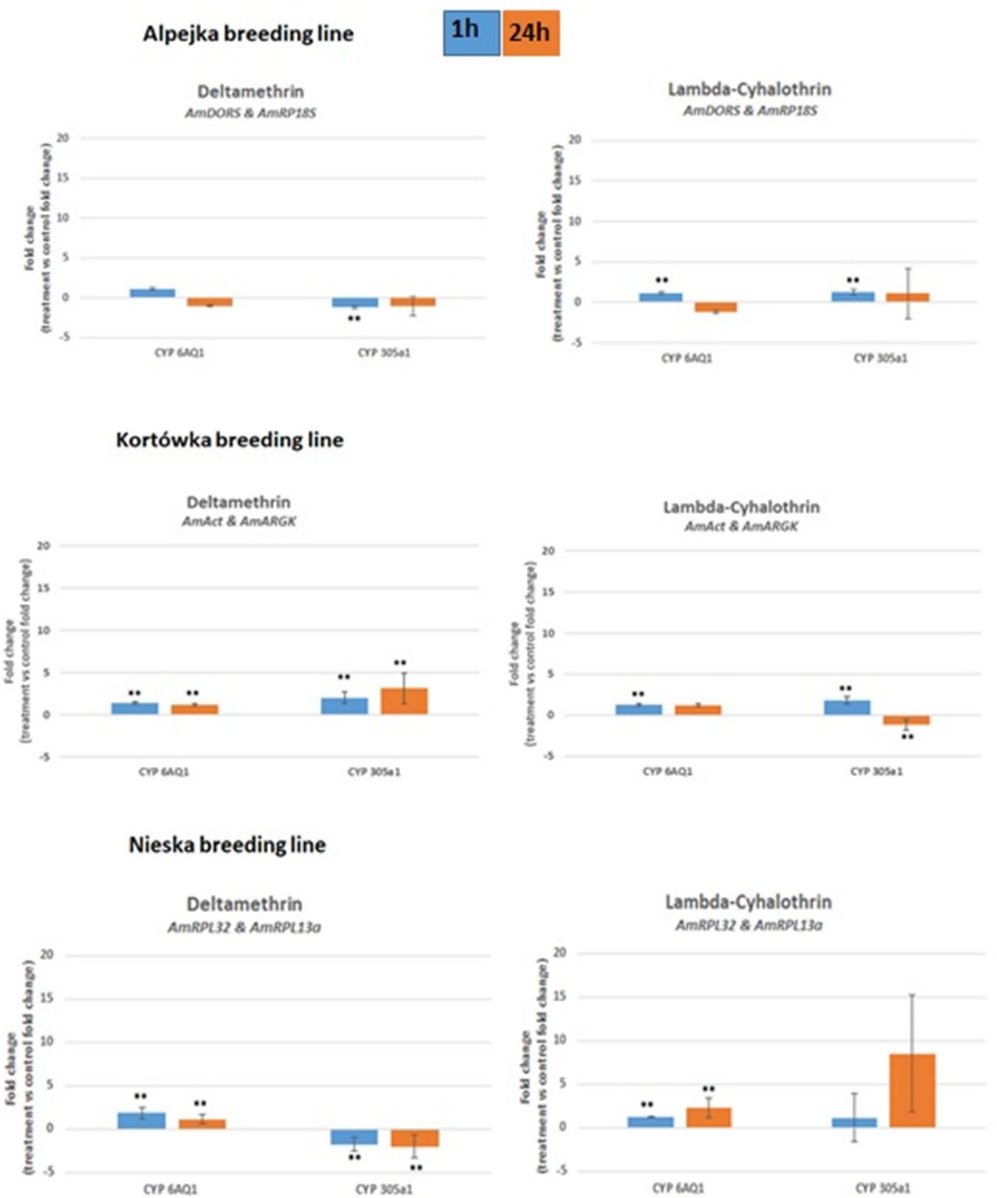
Expression of the two *AmCYP450* genes (*AmCYP6AQ1* and *AmCYP305a1*) in three breeding lines of *Apis mellifera* L., namely, Alpejka (**A**), Kortówka (**B**) and Nieska (**C**), treated with either deltamethrin or lambda-cyhalothrin, normalized against the indicated reference genes: (**A**) *AmDORS* and *AmRP18S*; (**B**) *AmAct* and *AmARGK*; (**C**) *AmRPL32* and *AmRPL13a*). Blue bars—1 h post treatment, orange bars—24 h post treatment. Error bars represent the standard deviation. Error bars represent the standard deviation. The Mann–Whitney U-test was used. **p < 0.01, *p < 0.05.

Next, the expression of *AmCYP450* genes was validated in each breeding line individually (Fig. 6). For the Kortówka line, the *AmAct* and *AmARGK* genes were chosen; for Alpejka, *AmDORS* and *AmRP18S* were used for normalization; and for the Nieska line, the *AmRPL32* and *AmRPL13a* genes were selected as the best normalizers for expression of the mentioned *AmCYP450s*.

When considering the expression of *AmCYP450s* in honeybees treated with pyrethroids, we observed that the expression of *AmCYP6AQ1* and *AmCYP305a1* in insects belonging to the Alpejka breeding line changed slightly. On the other hand, in the Kortówka breeding line, the level of expression of the *AmCYP305a1* gene increased slightly 1 h and 24 h after deltamethrin treatment. For the Nieska breeding line, the expression of *AmCYP6AQ1* was slightly upregulated in insects treated with both pyrethroids, whereas the expression of *AmCYP305a1* was downregulated in honeybees treated with deltamethrin. These data also showed that each breeding line of tested insects responded differently to pyrethroid treatment, taking into account changes in the expression level of the *AmCYP450s* genes tested (Fig. 6).

**Figure 7:**
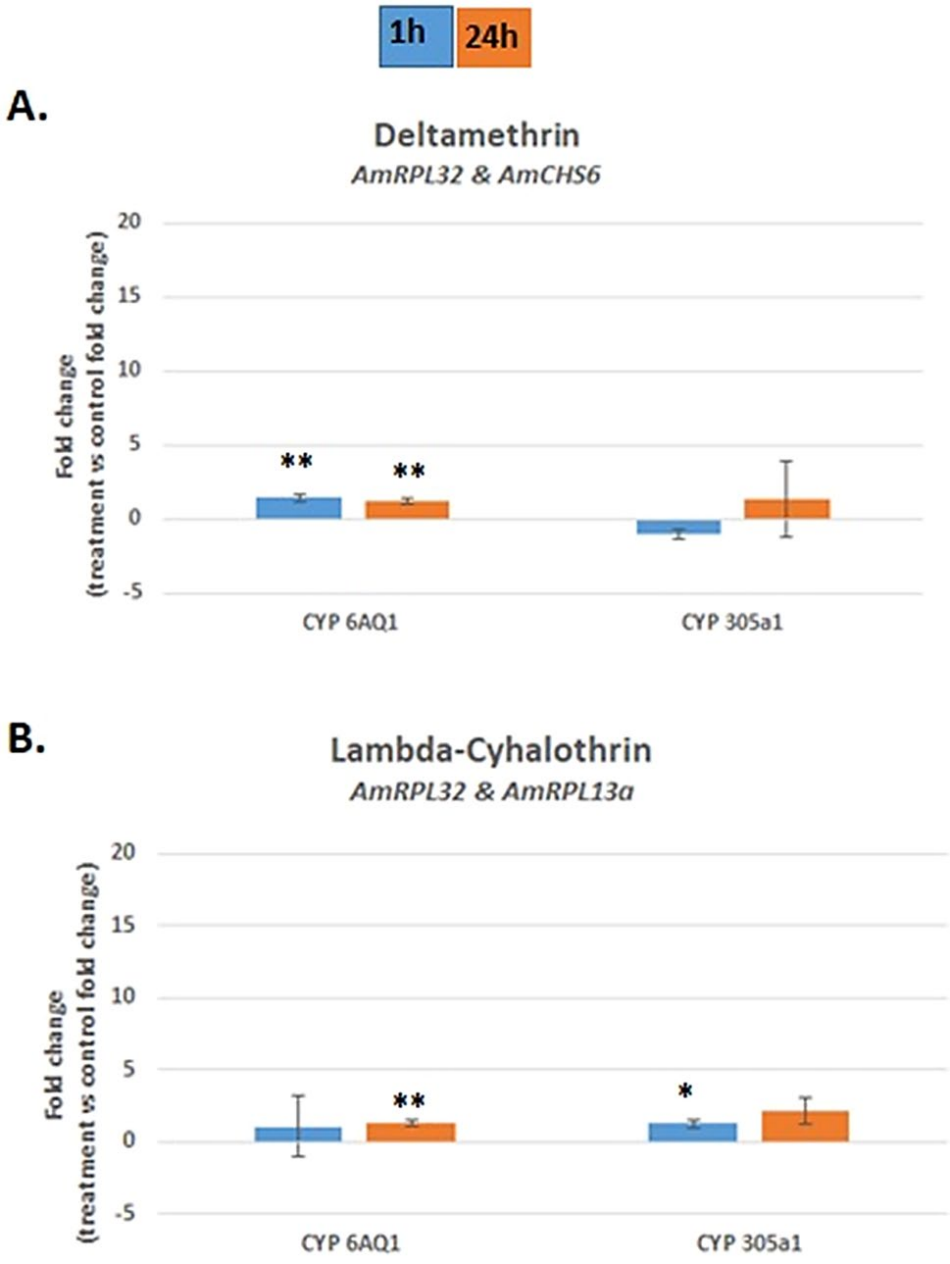
Expression of the two *AmCYP450* genes *AmCYP6AQ1* and *AmCYP305a1* in *Apis mellifera* L. treated with either deltamethrin (**A**) or lambda-cyhalothrin (**B**), normalized against the indicated reference genes: (**A**) *AmRPL32* and *AmCHS6*; (**B**) *AmRPL32* and *AmRPL13a*. The effects of the two active compounds used to treat honeybees were considered separately. Blue bars: 1 h post treatment, orange bars: 24 h post treatment. Error bars represent the standard deviation. Error bars represent the standard deviation. The Mann–Whitney U-test was used. **p < 0.01, *p < 0.05.

Finally, the expression levels of the *AmCYP6AQ1* gene and the *AmCYP305a1* gene were validated separately in insects under deltamethrin treatment and lambda-cyhalothrin treatment (Fig. 7, Table 6). The active substances used in our research model have little effect on changes in the expression level of the analysed *AmCYP450s* genes. In particular, when analysing the effect of deltamethrin on *AmCYP450* expression, a minor increase in the expression level of the *AmCYP6AQ1* gene 1 h post treatment and 24 h after pyrethroid exposure (1.40-fold change and 1.26-fold change, respectively, p < 0.01) was indicated. Accordingly, in lambda-cyhalothrin-treated insects, *AmCYP6AQ1* showed a modest, statistically significant increase in expression level 24 h after pyrethroid treatment (1.34-fold change, with p < 0.01). Similarly, the expression level of the *AmCYP305a1* gene was somewhat stable over time, reaching a 1.35-fold change (with p < 0.05) in bees 1 h after treatment with lambda-cyhalothrin (Fig. 7).

Additionally, changes in *AmCYP450s* expression levels normalized against two unstable HKGs were also analysed (see Supplementary Figs. S2, S3 and S4). The use of inappropriate normalizers in differential gene expression analysis resulted in increased statistical significance at the expense of an increased error range and changes in the expression levels of target genes in individual research models (e.g., for the Nieska line, the *AmCYP305a1* gene expression level 24 h after lambda-cyhalothrin treatment was almost 40 times higher than that obtained if the least stable genes were selected (see Supplementary Fig. S3). Moreover, the use of the highly unstable HKGs for validation gives different, highly discrepant results, as in the case of the Kortówka line, where after using the most stable genes, a decrease was observed in the level of *AmCYP305a1* gene expression (1.19-fold change), while using the least stable genes resulted in a 3.11-fold increase in the expression of a given gene (see Supplementary Fig. S3).

## Discussion

To minimize both biological and experimental errors in quantitative analyses performed by means of real-time qPCR, it is important to choose the most stable reference genes for normalization of RNA input. However, this requires an individualized research approach for each analysed parameter ^9,17^. One such parameter is the fitness and mortality of bees associated with commonly used insecticides, which has been extensively discussed^18–22^.

In this study, we investigated the expression stability of 14 candidate reference genes of *A. mellifera* L., belonging to Carnolian honeybees, exposed to pyrethroids. The selected subspecies of the honeybee was treated with two insecticides: deltamethrin^23^ and lambda-cyhalothrin^24^. It should be remembered that honeybees of various genetic background (like the three breeding lines described in the study: Alpejka, Nieska and Kortówka) might react differently at the level of insecticide sensitivity^25,26^ what can expressed at the molecular level.

Carnolian honeybees are highly adapted to nectar and climatic flow both in Poland and worldwide^27^. Analysis of all the obtained data described in this study on Carnolian honeybees under pyrethroid treatments indicated 5 stably expressed genes: *AmHMBS* (responsible for haem synthesis and porphyrin metabolism)*, AmCHS6* (responsible for synthesis of chitin)*, AmRPL32* (ribosomal protein gene)*, AmAct* (encoding cytoskeletal structural proteins)*,* and *AmTub* (encoding cytoskeletal structural proteins).

Analysis of the expression stability of selected candidate reference genes with respect to individual breeding lines distinguished a common high-scoring gene, *AmRPL13a,* in terms of stability for all the tested lines. On the other hand, *AmDORS*, *AmAct* and *AmTub* were selected as the most stable genes in the Alpejka, Kortówka and Nieska breeding lines, respectively. Similarly common most stable genes were also observed between the Kortówka and Nieska lines (the *AmARGK* gene) and between the Alpejka and Nieska lines (the *AmRP18S* gene). The expression level of the *AmARGK* gene does not change after carbon dioxide narcosis in honeybee workers^28^; however, it should be noted that the amount of ARGK protein in the antennae can vary between bee families^29^ . The ribosomal genes (from the functional rRNA-coding regions) are structurally conserved and homogeneous throughout the nuclear and mitochondrial genomes in honeybees^30^ and are often used as reference genes for differential expression studies^31–33^. In research on the effects of imidacloprid treatment on honeybees, ribosomal genes have been shown to be upregulated^13^, which means that they should be approached with caution as potential reference genes. The analyses also show the variable levels of expression of target genes relative to the *AmDORS* gene described in the literature^34^. Depending on the breeding line tested, the expression stability results for individual genes were classified slightly differently (Tables 3, 4, 5); therefore, in experiments, both the population and the breeding line should be determined with full accuracy to avoid statistical errors in research.

The stability ranking of HKGs in honeybees under pyrethroid treatment, when the active compounds (deltamethrin or lambda-cyhalothrin) were considered separately, showed three common most stable reference genes: *AmRPL32*, *AmTub* and *AmHMBS*. These results were confirmed with the data previously obtained when active substances were analysed together; however, it should again be noted that the genes were placed at different positions in the ranking order (after deltamethrin treatment: *AmRPL32, AmCHS6, AmTub, AmARGK* and *AmHMBS*; after lambda-cyhalothrin treatment: *AmRPL32, AmAct, AmRPL13a, AmHMBS* and *AmTub*). Such differences may occur due to differences in the sample sizes analysed individually. For the entire set of Carnolian honeybees, all data obtained in the experiments were taken into account. In turn, for the analysis of bees after treatment with deltamethrin or lambda-cyhalothrin, data obtained for a specific pyrethroid active substance treatment/exposure were taken (limiting the sample size from 108 bees up to 72 individuals). This is why the selection of the sample is such an important aspect of research related to differential gene expression^9^.

The validation of the indicated most stable reference genes showed that the selection of inappropriate normalizers, the expression of which is not stable under the conditions being tested, can significantly affect the final results of the analysis of the target gene of interest. The values may vary by up to 40 times, as was observed for the expression level of the *AmCYP6AQ1* gene in the Nieska line exposed to lambda-cyhalothrin (24 h after treatment), when we compared the results obtained by using the most stable genes and least stable genes for normalization (see Supplementary Fig. S3). The statistical significance and direction of changes in the level of expression between two time points were also divergent after the selection of relatively less stable reference genes for analysis (as was the case for the Nieska breeding line, as stated earlier) (see Supplementary Figs. S2, S3, S4). Therefore, optimization of testing data by using various statistical programs is very important when studying changes in the expression of target genes relative to that of a reference gene^12^. It should also be noted that the selection of HKGs may differ if the research model assumes testing on populations, not on specific breeding lines, as presented in Tables 2, 3, 4, 5. Previous work also showed that the differences in the expression stability results for reference genes may be due to the season in which the study was conducted^35^ and the stage of maturation of the tested individuals^36^. The expression levels of the *AmCYP450* genes validated against the selected HKGs confirmed some behavioural observations for the developmental lines tested. Namely, slight changes in the expression of the *AmCYP6AQ1* and *AmCYP305a1* genes were observed for the Alpejka line (Fig. 6), in which individuals showed the highest liveliness among the three breeding lines during the experiment. Accordingly, the most considerable increase in the expression of these genes was demonstrated for the Nieska line (Fig. 6), the individuals of which tended to gather in groups and exhibited low activity (data not shown).

To summarize, regardless of the experimental conditions or tested breeding line of the examined insects, the above studies indicated the three following HKGs as reference genes to be considered, as they were classified by each analysis as being the most stable genes: *AmRPL32, AmAct* and *AmRPL13a*.

## Methods

### Insects used in the study

In this study, three breeding lines of *A. mellifera* L. were used, namely, Kortówka, Alpejka and Nieska, all belonging to Carniolan honeybees. The insects were taken from original hives by a beekeeper and were individually treated with a 1 µl dose of one of the following pyrethroids: deltamethrin (0.75 ml/L (4.8%)) or lambda-cyhalothrin (0.75 ml/L (4.81%)). Non-treated insects were used as a control. Then, the treated bees were gathered (6–15 insects, whereas for RNA isolation 6 insects were taken) in bee cages and collected 1 h and 24 h after treatment. During this time, the insects were kept at room temperature on a laboratory bench. Then, the insects were immediately frozen in liquid nitrogen and stored at -80 °C.

### RNA isolation and cDNA synthesis

Single insects (from six biological replicates) were pulverized in liquid nitrogen using a mortar and pestle and were subsequently stored at − 80 °C for further analyses. Next, up to 100 mg of pulverized material was taken for total RNA extraction. RNA isolation was performed using 1 ml of TriReagent Solution (Invitrogen) followed by RNA precipitation with propanol. The resulting RNA pellet was washed with 70% ethanol, air-dried and resuspended in nuclease-free water. The concentration of the RNA, as well as its purity (the 260/230 and 260/280 values) were measured using a NanoDrop 2000 spectrophotometer (Thermo Scientific), whereas the quality of the RNA was assessed by means of gel electrophoresis.

Contaminant genomic DNA in the RNA samples was removed using dsDNase enzyme (Thermo Scientific). Next, cDNA synthesis was performed using the Maxima First Strand cDNA Synthesis Kit for RT-qPCR (Thermo Scientific) and 3 μg of total RNA. The resulting cDNA was finally diluted 3 times with water (50 ng/μl).

### Primer selection and real-time quantitative PCR

The primers used in this study were designed using Primer3 online software^37,38^. The coding sequences of target transcripts were retrieved from GenBank and further analysed with Primer3 software to indicate the best pairs for RT-qPCR (Table 1). The selected primers were tested for their specificity: initially, all the tested sequences were verified by BLAST, and next, the primers were used in subsequent end-point RT-PCR to check the estimated size of the resulting amplicons. The RT-PCRs were performed in 20 μl reactions containing 1 × reaction master mix (DreamTaq PCR Master Mix, Thermo Scientific), 0.5 μM forward primer, 0.5 μM reverse primer and 1 μl of cDNA. The reaction was incubated for 3 min at 95 °C, followed by 35 cycles of 95 °C for 20 s, 60 °C for 30 s, and 72 °C for 30 s. After incubation at 72 °C for an additional 10 min, the reactions were resolved on a 1% agarose gel, and the PCR products were gel-purified and subsequently cloned into *Escherichia coli* DH10B using the CloneJET PCR Cloning Kit (Thermo Scientific). The recombinant plasmids were isolated from transformed bacteria, and the inserted cDNAs were sequenced by Genomed (Warsaw, Poland).

RT-qPCR was performed as follows: the 10 μl reaction mixture contained 1 × master mix (iTaq Universal SYBR Green Supermix, Bio-Rad), 0.5 μM forward primer, 0.5 μM reverse primer and 1 μl of cDNA. The reaction was incubated for 3 min at 95 °C, followed by 40 cycles of 20 s at 95 °C, 30 s at 60 °C, and 30 s at 72 °C. After the last cycle, a melting curve was generated by increasing the temperature from 60 °C to 95 °C. RT-qPCR was performed in three technical replicates using the real-time PCR system (QuantStudio5, Thermo Scientific).

### Statistical analysis of HKGS and validation

Selection of the best reference genes was performed using previously described calculation algorithms, namely, geNorm^17^, BestKeeper^39^, NormFinder^40^ and the ΔCT method^41^. The detailed description of the methods was indicated in Supplementary File.

## Supplementary information

Supplementary Information.
