# Supplementary material for: Evaluation of the expression stability of reference genes in Apis mellifera under pyrethroid treatment
Source: Sci Rep. 2020 Sep 30;10:16140. doi: 10.1038/s41598-020-73125-w (PMC7527991; doi:10.1038/s41598-020-73125-w)
Supplement: Supplementary file 1 — Supplementary Information. [file 41598_2020_73125_MOESM1_ESM.pdf]

Evaluation of the expression stability of reference genes in *Apis mellifera* under pyrethroid treatment

Przemysław Wieczorek, Patryk Frąckowiak and Aleksandra Obrępalska-Stęplowska*

Department of Molecular Biology and Biotechnology, Institute of Plant Protection-National Research Institute, Władysława Węgorka 20 St., 60-318 Poznań, Poland

*Corresponding author: olaob@tlen.pl

SUPPLEMENTARY METHODS

STATISTICAL ANALYSIS OF HKGS AND VALIDATION

The data from qPCR (Ct values) have been organised in table and loaded to R software. For the geNorm expression stability analysis the M value of each housekeeping gene was calculated (using NormqPCR package), whereas, for the BestKepeer (from ctrlGene package) the gene stability was assessed based on the standard deviation (SD) of Ct values. The BestKeeper determines the geometric mean of the Cq values of the genes and standard deviation (SD), therefore lower SD values indicate more stable genes. In addition, it calculates the correlation (R2) of each candidate gene with other genes, following which, highly correlated candidate genes are combined to evaluate P values. To estimate both intragroup and intergroup variation (these two variation values where combined to give stability value) the NormFinder software add-in for GenEx ver 6 software (MultiD Analyses) has been used. The lowest value indicating the most stable gene expression. The delta Ct method compares differences of Ct values by comparing two reference genes pairwise. The candidate reference genes are ranked according to the mean standard deviation of the mean delta Ct differences. RefFinder integrates all four programs (geNorm, NormFinder, BestKeeper, and Delta Ct method) and calculates a weighted geometric mean for a ranking of the best reference genes.

To determine the optimal number of HKGs was also calculated in geNorm software using the pairwise variation (Vn/n+1) between the two sequential normalization factors (NFn and NFn+1) with the cut-off threshold of 0.15.

The statistical analysis for normalisation of selected HKGs was performed using GenEx ver 6 software. The descriptive statistic have been used to determine normal distribution in each tested group (control, delthamethrin treatment and lambda-cyhalothrin treatment after isolation in 1 and 24 hours post treatment). The Mann-Whitney U-test was used to compare differences

between two groups and a value of $p<0.05$ was considered to indicate a statistically significant difference in validation process.

SUPPLEMENTARY FIGURES

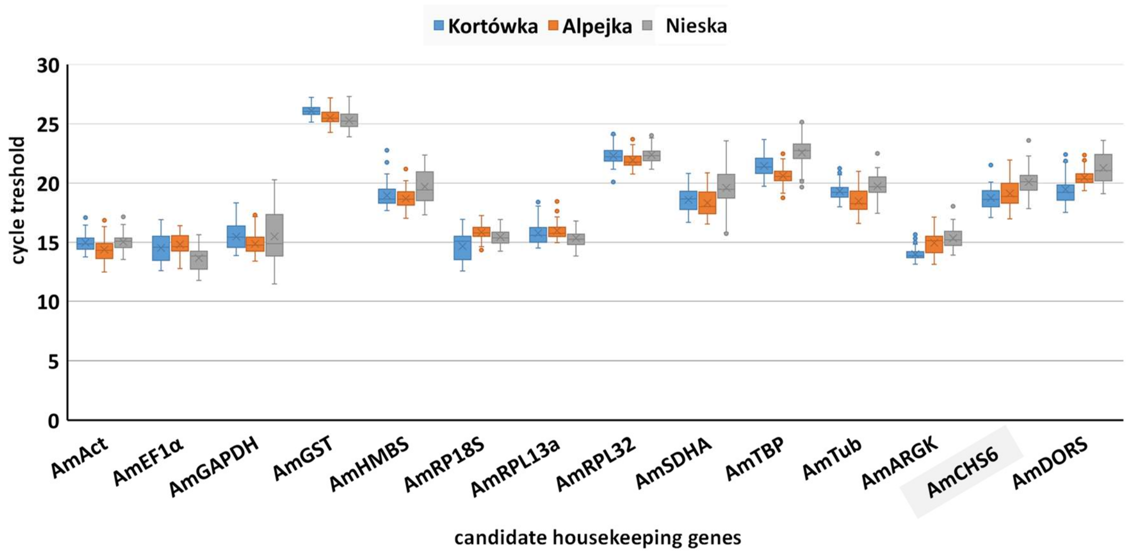

Supplementary Figure S1. Box plot indicating the distribution of C_T values after RT-qPCR for each primer pair. The C_T values were considered among the three tested breeding lines (Kortówka, Alpejka and Nieska).

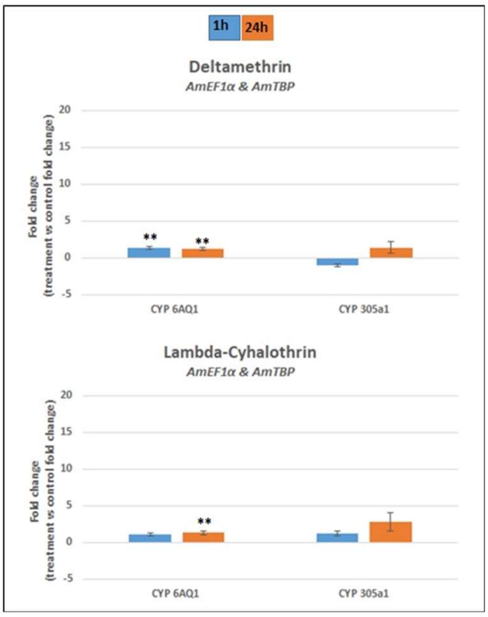

Supplementary Resource S2. Expression of the two *AmCYP450* genes *Am*C*YP6AQ1* and *AmCYP305a1* in *Apis mellifera* treated with either deltamethrin (A) or lambda-cyhalothrin (B), normalized against the relatively less stable reference genes (*AmEF1α* and *AmTBP*). Blue bars: 1 h post treatment, orange bars: 24 h post treatment. Error bars represent the standard deviation. The Mann-Whitney U-test was used. **p<0.01, *p<0.05.

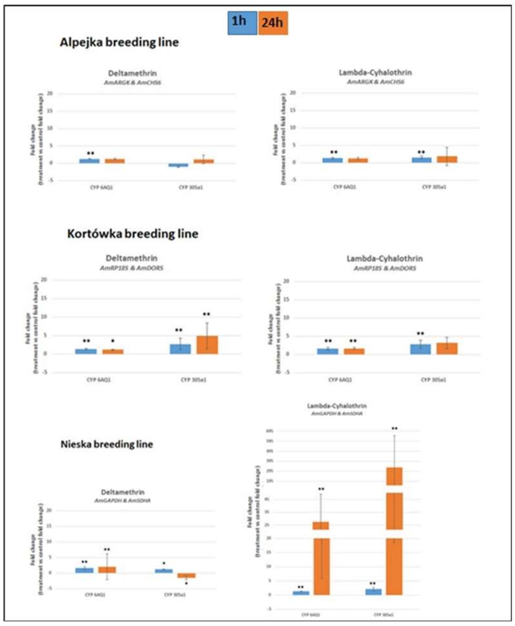

Supplementary Figure S3. Expression of the two *AmCYP450* genes *AmCYP6AQ1* and *AmCYP305a1* in three breeding lines of *Apis mellifera*, namely, Alpejka (A), Kortówka (B) and Nieska (C), treated with either deltamethrin or lambda-cyhalothrin, normalized against the relatively less stable reference genes: (A) *AmARGK* and *AmCHS6*; (B) *AmRP18S* and *AmDORS*; (C) *AmGAPDH* and *AmSDHA*. Blue bars: 1 h post treatment, orange bars: 24 h post treatment. Error bars represent the standard deviation. Error bars represent the standard deviation. The Mann-Whitney U-test was used. **p<0.01, *p<0.05.

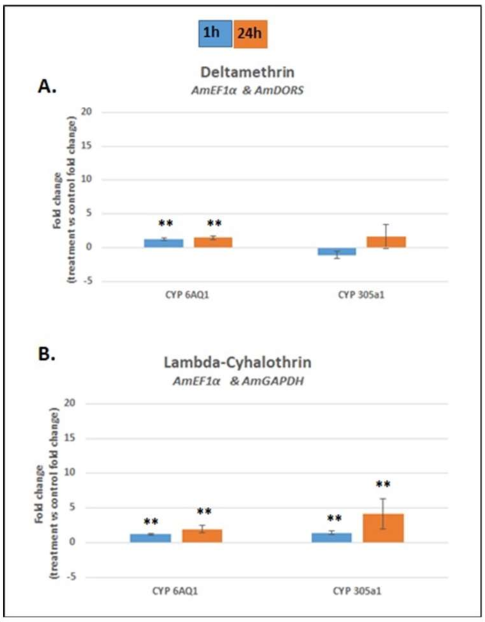

Supplementary Figure S4. Expression of the two *AmCYP450* genes *AmCYP6AQ1* and *AmCYP305a1* in *Apis mellifera* treated with either deltamethrin (A) or lambda-cyhalothrin (B), normalized against the relatively less stable reference genes: (A) *AmEF1α* and *AmDORS*; (B) *AmEF1α* and *AmGAPDH*. The effects of the two active compounds used to treat honeybees were considered separately. Blue bars: 1 h post treatment, orange bars: 24 h post treatment. Error bars represent the standard deviation. The Mann-Whitney U-test was used. **$p<0.01$, *$p<0.05$.
